# Grafting vigour is associated with DNA de-methylation in eggplant

**DOI:** 10.1038/s41438-021-00660-6

**Published:** 2021-11-01

**Authors:** Elisa Cerruti, Carmina Gisbert, Hajk-Georg Drost, Danila Valentino, Ezio Portis, Lorenzo Barchi, Jaime Prohens, Sergio Lanteri, Cinzia Comino, Marco Catoni

**Affiliations:** 1 Department of Agricultural, Forest and Food Sciences, Plant Genetics and Breeding, University of Torino, Grugliasco, Italy; 2 The Sainsbury Laboratory, University of Cambridge, Cambridge, UK; 3 Institute for Conservation & Improvement of Valencian Agrodiversity (COMAV), Universitat Politècnica de València, Valencia, Spain; 4 Computational Biology Group, Department of Molecular Biology, Max Planck Institute for Developmental Biology, Tübingen, Germany; 5 School of Biosciences, University of Birmingham, Birmingham, United Kingdom; 6 Institute for Sustainable Plant Protection, National Research Council of Italy, Torino, Italy

**Keywords:** Plant development, DNA methylation

## Abstract

In horticulture, grafting is a popular technique used to combine positive traits from two different plants. This is achieved by joining the plant top part (scion) onto a rootstock which contains the stem and roots. Rootstocks can provide resistance to stress and increase plant production, but despite their wide use, the biological mechanisms driving rootstock-induced alterations of the scion phenotype remain largely unknown. Given that epigenetics plays a relevant role during distance signalling in plants, we studied the genome-wide DNA methylation changes induced in eggplant (*Solanum melongena*) scion using two interspecific rootstocks to increase vigour. We found that vigour was associated with a change in scion gene expression and a genome-wide hypomethylation in the CHH context. Interestingly, this hypomethylation correlated with the downregulation of younger and potentially more active long terminal repeat retrotransposable elements (LTR-TEs), suggesting that graft-induced epigenetic modifications are associated with both physiological and molecular phenotypes in grafted plants. Our results indicate that the enhanced vigour induced by heterografting in eggplant is associated with epigenetic modifications, as also observed in some heterotic hybrids.

## Introduction

Grafting is the process of joining plant tissues of two plants: the scion (upper part) and rootstock (lower part including roots), which then continue to grow together combining the favourable characteristics of the genotypes involved. Plant grafting is a naturally occurring process, but it was systematically used by humans as an agricultural technique to improve the cultivation of fruit trees. Over centuries, grafting allowed humans to facilitate tree propagation, reduce juvenility, provide resistance to biotic and abiotic stresses and control plant growth^1^. Starting from the early 20th century, the use of grafting was extended to vegetables, in particular Solanaceae and Cucurbitaceae species, also in interspecific combinations (i.e. with scion and rootstock belonging to different species) to improve yield^2^. Although not directly involved in fruit production, rootstocks are selected for their ability to regulate salinity and drought tolerance, water-use efficiency and nutrient uptake, soil-borne pathogen resistance, scion vigour and architecture, mineral element composition, fruit quality and yield in a broad range of species^3–5^. While some of these aspects can be explained by intrinsic properties of rootstock genotypes, the molecular mechanisms at the base of the rootstock-mediated control of scion phenotypes remain mostly unknown.

So far, several studies on model species have demonstrated that a possible molecular mechanism involved in grafting is a bidirectional long-distance transport of macromolecules, such as mRNAs transcripts, microRNAs (miRNAs) and other small RNAs (sRNAs). These RNAs are able to trigger physiological changes through the graft junction^6–10^ which might also result in the emergence of vigour. Using grafting as an experimental system, it was found that sRNAs are able to induce epigenetic variation through an RNA-directed DNA methylation (RdDM) pathway in recipient tissues, mostly altering cytosines methylation in CHH context (H = adenine, thymine or cytosine)^7,8,11^. Although these findings provided a first molecular basis for grafting-induced changes in organ growth and development they lack the exact mechanism driving rootstock-dependent physiological effects in grafted plants.

Here, we introduce the eggplant (*Solanum melongena* L., 2n = 2x = 24) as a model system to study general molecular mechanisms of grafting. Eggplant (also known as aubergine), is one of the most common commercially grafted solanaceous crops^12^. Several rootstocks were selected to improve the quality of eggplant cultivation, providing resistance/tolerance to soil pathogens and inducing vigorous growth of the scions^13,14^. Commercial grafting of eggplants also makes large use of interspecific combinations to increase plant vigour and resistance to pathogens, and the most popular rootstocks include wild solanaceous species, like the eggplant wild relative *Solanum torvum* Sw. (Turkey berry), or tomato hybrids developed specifically for being used as rootstock^15,16^. A recent study provided the first evidence of locus‐specific changes in DNA methylation in interspecies grafting of *S. melongena* and other Solanaceae^17^, suggesting that rootstock-induced epigenetic alterations can produce physiological changes in eggplant scions.

Here we analysed the genome-wide methylation profiles of eggplant scions from interspecific grafting combinations using *S. torvum* and a tomato hybrid as rootstocks. We observed that the enhanced vigour induced by these rootstocks is associated with a genome-wide decrease of CHH methylation, occurring at both coding genes and transposons. In addition, we found that DNA demethylation is also associated with changes in transcription between hetero-grafted and self-grafted scions. We found that many regulated genes were involved in plant developmental processes related to the grafting response and for transposable elements (TEs) transcriptional regulation appeared to be modulated in an age-related fashion.

## Results

### Enhanced vigour in hetero-grafted eggplant scions is associated to genome-wide CHH hypomethylation

**Fig. 1:**
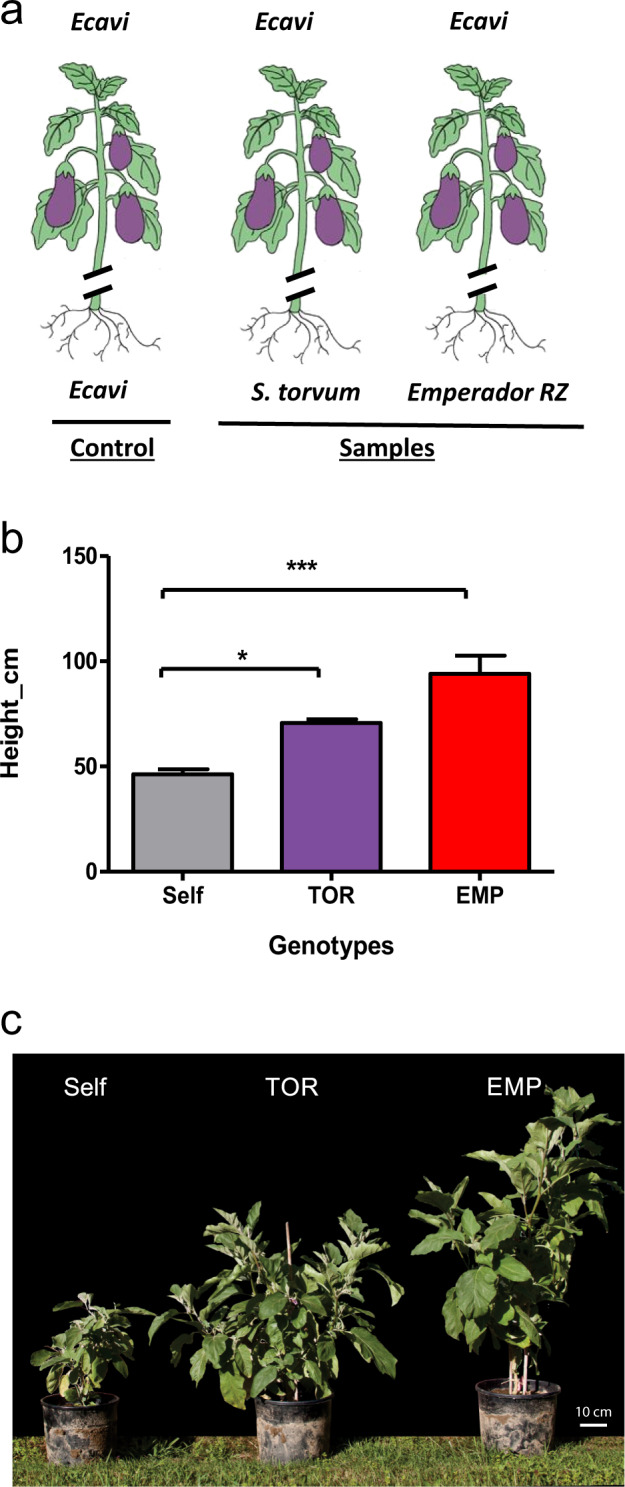
Heterografting induces vigour in eggplant scions. **a** Representation of grafting experimental design considering the conditions under investigation (from left to right): (1) self-grafted eggplant (var. ‘Ecavi’) scions, (2) ‘Ecavi’ scions onto *S. torvum* rootstocks (TOR) and (3) ‘Ecavi’ scions onto tomato ‘Emperador RZ’ rootstocks (EMP). **b** Height differences between hetero-grafted and self-grafted plants were observed 5 months after grafting occurred. From left to right eggplant scions grafted on ‘Ecavi’ eggplant (Self), *S. torvum* (TOR) and tomato ‘Emperador RZ’ (EMP). Asterisks mark statistically significant differences (ANOVA one-way, *P* < 0.05). Error bars represent the SD of three replicates. **c** Picture displaying differences in vigour between plants representative of the grafting conditions (Self, TOR and EMP) at 5 months post grafting

To study the effect of grafting on vigour, we grafted eggplant scions (double haploid [DH] line derived from the commercial hybrid ‘Ecavi’) on three rootstocks: (i) the wild species *Solanum torvum* (TOR), (ii) the tomato F_1_ commercial hybrid ‘Emperador RZ’ (EMP) and (iii) the same eggplant genotype (self-grafting) (Fig. 1a). Both *S. torvum* and ‘Emperador RZ’ were previously reported to induce vigour in eggplant scions^13,15^. Indeed, 5 months after grafting, the hetero-grafted plants showed a remarkable and statistically significant increase in height when compared to self-grafted eggplants, used as reference control (Fig. 1b). Depending on whether the rootstock used was *S. torvum* or the tomato ‘Emperador RZ’, eggplant scions respectively displayed a marked bushy phenotype or more pronounced vertical growth (Fig. 1c).

It has been hypothesised that DNA methylation is the driving mechanism generating phenotypic diversity via grafting^18^. To test this hypothesis, we studied whether changes in cytosine methylation might indeed be associated with observed differences in scion vigour. We performed genome-wide bisulfite sequencing of DNA samples extracted from two eggplant scions of each grafting combination described above, and two biological replicates of ungrafted eggplants. After quality control and data processing, roughly 80 M reads per sample were sequenced, with an average 10X coverage of the eggplant genome. The results obtained from ungrafted plants allowed us to generate the first eggplant genome methylation profile at single cytosine resolution, which in leaf tissue displayed 91% methylation in CG, 84% in CHG and 19% in CHH contexts (Table S1). Moreover, in these ungrafted plants, the DNA methylation levels in CG and CHG contexts were more pronounced in the central part of each chromosome, while decreased levels were found at the terminal parts of the chromosome arms. In contrast, CHH methylation levels were more evenly distributed across the genome (Fig. S1). This profile is similar to DNA methylation patterns reported for the same tissue in other Solanaceae^19,20^ where a general anti-correlation between DNA methylation (mostly in CG and CHG context) and gene density is observed (Fig. S1), which is associated with an increasing abundance of methylated TEs in the central part of chromosomes.

**Fig. 2:**
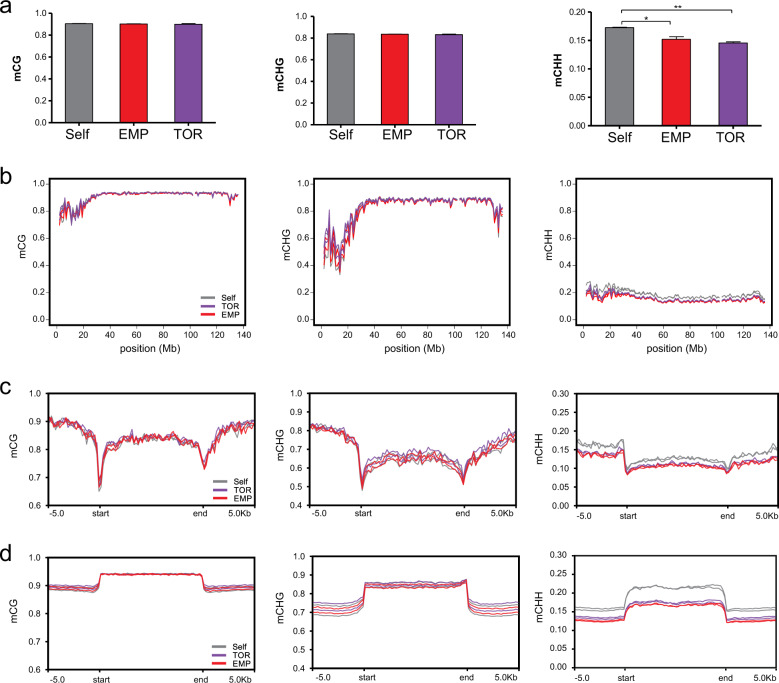
Heterografting is associated to CHH genome hypomethylation. **a** Methylation averaged at all cytosines for two replicates per hetero-grafted condition. Error bars represent SD (*n* = 2). *t*-test *p* value: * = 0.0005, ** = 0.0007. **b** Distribution of DNA methylation at the three cytosine contexts (mCG, mCHG and mCHH) along chromosome 1 of the eggplant genome, in scions self-grafted (Self), grafted on *S. torvum* (TOR) or grafted on tomato ‘Emperador RZ’ (EMP). Results for the remaining chromosomes (2 to 12) are displayed in Fig. S2. **c** Distribution of averaged DNA methylation at annotated genes in the three contexts (mCG, mCHG and mCHH,) in the eggplant scions self-grafted (Self), grafted on *S. torvum* (TOR) or grafted on tomato ‘Emperador RZ’ (EMP). **d** Distribution of averaged DNA methylation level at annotated repetitive elements, description analogous to (**c**)

While the methylation profile of self-grafted scions was similar to ungrafted plants (Table S1), when we investigated methylation profiles in hetero-grafted scions we observed a significant genome-wide decrease in CHH methylation of 3.37 and 2.58%, respectively in scions grafted onto *S. torvum* and ‘Emperador RZ’ when compared to the self-grafted plants (Fig. 2a). This decrease appeared to be uniformly distributed along chromosomes (Fig. 2b and Fig. S2). Unlike methylation in the CHH context, the methylation in CG and CHG contexts remained unchanged in both self- and hetero-grafted scions (Fig. 2a, b and Fig. S2). Further analyses showed that CHH hypomethylation was more prominent at TEs than at coding genes, but not specific for a particular TE family (Fig. 2c, d and Fig. S3).

To explore the nature of observed DNA demethylation changes, we screened for differentially methylated regions (DMRs) in the CHH context, separately in scions grafted on *S. torvum* and in scions grafted on ‘Emperador RZ’. As a control, we used self-grafted scion replicates as common denominator. Consistent with the general methylation profiles observed in our previous experiment, the large majority (>98%) of CHH DMRs identified were hypomethylated in the hetero-grafted scions (Fig. S4a and Tables S2, S3). The distribution of these CHH DMRs were very similar in both hetero-grafted scions, and appeared evenly distributed across the genome (Fig. S4b, c). We then determined how many CHH DMRs overlap with annotated genomics features. We observed that DMRs in both hetero-grafted scions were significantly enriched in Class II TEs (*p* values <10^−100^) and in gene promoters (*p* values <10^−18^), when compared against a set of randomly selected DNA regions (Fig. S4d).

### Hetero-grafted plants display similar transcriptional profiles

**Fig. 3:**
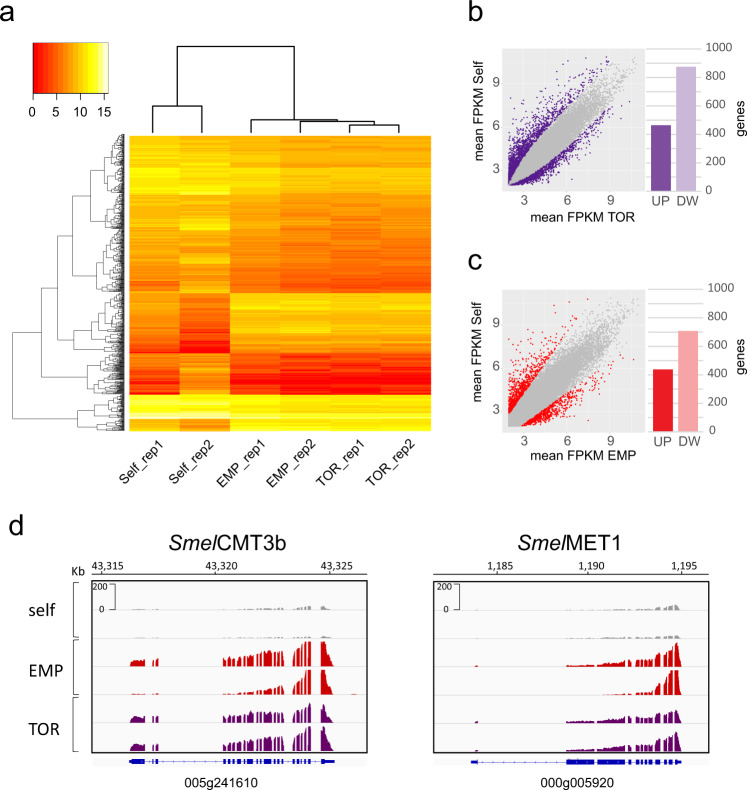
Hetero-grafted scions display similar gene expression profiles. **a** Heatmap illustrating the expression (log2 FPKM) of differentially expressed genes (FDR <0.05) in hetero-grafted plants and self-grafted controls. Each row represents one gene (*n* = 773). Coloured bars at the top-left indicate the expression level. Each column represents an eggplant scion grafted on different rootstock (TOR = *S. torvum*, EMP = tomato ‘Emperador RZ’ and Self = self-grafted). Expression values were ordered according to hierarchical clustering (hclust and heatmap2 in an R software environment). The Euclidean distance dendrogram is presented on the left. **b** Scatter-plots of annotated genes (*n* = 34,917) in Self and TOR conditions, each dot represents a gene and in purple are displayed differentially expressed genes between the two conditions. The bar plot on the right displays the number of upregulated (UP) and downregulated (DW) genes. **c** Scatter-plots of annotated genes in Self and EMP conditions. In red are displayed differentially expressed genes between the two conditions. The bar plot on the right displays the number of upregulated (UP) and downregulated (DW) genes. **d** Genome browser image showing the read coverage for eggplant *Smel*CMT3b and *Smel*MET1 genes. The gene names and structures are displayed below each graph

To further investigate whether differences in DNA methylation were associated with changes in transcription, we profiled the genome-wide RNA expression in the same grafted scion samples, by strand-specific RNA-sequencing (Table S4). Next, we compared the transcriptome of both eggplant hetero-grafted scions to self-grafted scions. Despite the use of different species as rootstock, we observed that the transcription profiles of eggplant scions grafted onto *S. torvum* and ‘Emperador RZ’ clustered together and clearly diverged from self-grafted controls (Fig. 3a). This indicates that both changes in DNA methylation and transcription levels are associated with the altered phenotype observed in hetero-grafted eggplants compared to self-grafted plants. Our differential expression analysis revealed a prevalence of downregulated genes in scions grafted onto both *S. torvum* (65%, Fig. 3b) and ‘Emperador RZ’ (61%, Fig. 3c). In particular, we observed that 464 genes were upregulated and 875 were downregulated in scions grafted onto *S. torvum*, while 434 and 704 genes were found respectively up and downregulated in scions grafted onto ‘Emperador RZ’ (Fig. S5a, b and Tables S5, S6). In addition, 151 upregulated and 462 downregulated genes are shared between the two hetero-grafted categories (Fig. S5a, b and Tables S3, S4). Validation performed by qPCR confirmed the change of expression levels of 11 selected genes (taken randomly among the differentially regulated), in scions grafted onto *S. torvum* (six genes) and onto ‘Emperador RZ’ (five genes) (Fig. S6 and Table S7). Next, we explored whether the decrease in CHH methylation observed in the two hetero-grafted conditions was associated with a change in the expression level of key DNA methyltransferase and demethylase enzymes previously characterised in eggplant^21^ (transcriptional status shown in Table S8). While none of the DNA demethylases significantly changed expression, we observed that the methyltrasferases *Smel*CMT3b (SMEL_005g241610) and *Smel*MET1 (SMEL_000g005920) were upregulated in both eggplant scions grafted on *S. torvum* and ‘Emperador RZ’, compared to the self-grafted controls (Table S8 and Fig. 3d), suggesting that epigenetic regulation could be differentially modulated in hetero-grafted plants. In order to examine whether differentially expressed genes (DEGs) were involved in specific developmental processes that could explain the vigour of hetero-grafted scions, we performed a GO enrichment analysis taking into account, separately, two datasets containing respectively up- and down-regulated genes in eggplants grafted on *S. torvum* and ‘Emperador RZ’ using the ShinyGO tool^22^ (Tables S9, S10). In both hetero-grafted scions, we observed enrichment (*p* value <0.05) among upregulated genes involved in early developmental processes such as cell division, regulation of cell cycle and DNA replication (Table S9 and Fig. S7), which is consistent with the increase of hybrid vigour^23,24^. In addition, the enrichment analysis on downregulated genes (*p* value <0.05) highlighted a basal response characterised by prevalent GO terms associated to transmembrane transport, ion binding and response to stimuli, which might be directly or indirectly triggered by grafting (Table S10 and Fig. S7). These results suggest that the enhanced vigour of hetero-grafted scions could be the direct consequence of transcriptional changes, thereby supporting the hypothesis that DNA methylation is the driving mechanism generating phenotypic diversity via grafting^18^. However, since we found little overlap of DMRs and DNA sequences of DEGs (Fig. S8), it is probable that most of the epigenetic changes are not directly associated with the regulation of gene expression, but rather hint towards grafting-induced mechanisms targeting only particular genes.

### Grafting modulates TEs expression

**Fig. 4:**
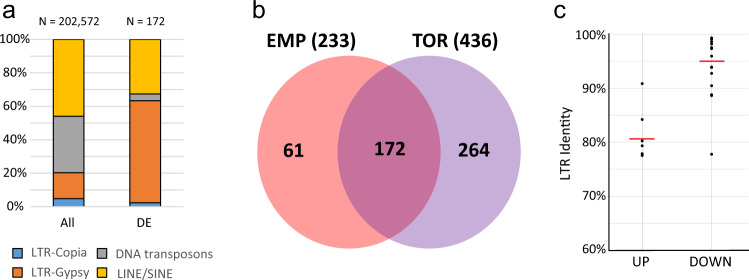
LTR-TEs regulation during heterografting. **a** Bar plot displays the distribution of families of TE differentially expressed (DE) in hetero-grafted combinations (compared to self-grafted controls). The composition of TE families for all annotated elements is reported as a comparison (All). **b** Venn-diagram displaying TEs differentially expressed shared between the scions grafted onto *S. torvum* (TOR) and tomato ‘Emperador RZ’ (EMP), data in parenthesis indicate the total number of differentially expressed TEs in each condition. **c** LTR identity values of de novo annotated intact LTR-TEs commonly regulated in both hetero-grafted scions, separated in upregulated (UP) and downregulated (DOWN) elements. The red lines represent averaged values (arithmetic mean)

We then inspected whether the transcriptional activity of TEs, which are directly silenced by DNA methylation, differed between hetero-grafted and self-grafted plants. As an initial approach we selected TE annotated sequences on the eggplant reference genome^25^. Next, we filtered only elements differentially regulated in at least one of the two hetero-grafted conditions (Table S11). TEs appeared to be regulated similarly to genes, with more downregulated elements (341 and 201 TEs downregulated and 95 and 32 TEs upregulated in scions grafted respectively onto *S. torvum* and ‘Emperador RZ’) compared to the self-grafted plants (Table S12). Specifically, we observed that retrotransposons belonging to the RTE class were the most abundant among upregulated TEs in scions grafted both onto *S. torvum* (53 TEs) and ‘Emperador RZ’ (16 TEs). On the other hand, the most represented downregulated TEs were LTR-TEs of the Gypsy superfamily, (106 and 119 TEs in ‘Emperador RZ’ and *S. torvum* hetero-grafted plants, respectively) of which, a significant proportion (105 TEs) were commonly repressed in both hetero-grafted scions **(**Fig. 4a, b and Table S12**)**. Interestingly, Gypsy is the most abundant group of TEs belonging to Class II, which have been found to be the most strongly hypomethylated in our DMR analysis (Fig. S4d). Therefore, to further investigate the link between LTR-TEs expression and DNA methylation we developed the functional annotation pipeline *LTRpred*^26^ and applied it to the eggplant genome assembly to de novo re-annotate LTR-TEs. We designed *LTRpred* to screen for old and young LTR-TEs and to predict their functional capacity based on well-defined sequence composition and intact sequence motifs. Together, *LTRpred* allowed us to study the association between novel LTR-TEs and their epigenetic regulation during grafting. Differential expression analysis of these newly annotated LTR-TEs again correlated in both hetero-grafted combinations (Figs. S5c, d and S9), similarly to what we previously observed for genes (Fig. 3). A downregulation trend was observed for the annotated LTR-TEs both in plants grafted onto *S. torvum* (73%) and ‘Emperador RZ’ (66%) compared to the self-grafted controls (Tables S13, S14). Specifically, in eggplant scions grafted onto *S. torvum*, 63 differentially expressed LTRs were identified (17 are upregulated and 46 downregulated) (Fig. S5c, d), while 32 TEs were differentially expressed in plants grafted onto ‘Emperador RZ’ rootstock (11 upregulated and 21 downregulated) (Fig. S5c, d). A significant proportion of these LTR-TEs (6 upregulated genes and 20 downregulated) were shared between the two heterograft combinations (Fig. S5c, d and Tables S13, S14**)**.

Interestingly, we observed that the average LTR identity, a strong indicator of the age of TEs, is high (=young elements) in LTR-TEs downregulated in hetero-grafted scions and consistently lower (=old elements) in upregulated LTR-TEs (Fig. 4c). However, considering that in hetero-grafted conditions both up- and down-regulated LTR-TEs appear to be analogously hypomethylated (Fig. S10a, b), our results suggest that this age-dependent regulation of LTR-TE expression cannot solely be explained by the level of DNA methylation, but seems to incorporate additional factors triggered by indirect epigenetic effects.

## Discussion

Eggplant is one of the most successful commercially grafted herbaceous plants, with a high degree of compatibility for interspecific grafting which may provide enhanced vigour and resistance to pathogens^12^. While most resistance to root pathogens of grafted plants derives from intrinsic properties of the rootstocks, the molecular mechanism of grafting and how particular graft combinations enhance scion vigour is largely unknown.

Grafting experiments in the model plant *Arabidopsis* have revealed that during long-distance movements, transgene-derived and endogenous sRNAs move across a graft union and are able to direct DNA methylation in the genome of the recipient cells, inducing physiological changes^9,18^. Here, using eggplant as a model for grafting-induced vigour, we found that genome-wide CHH hypomethylation in the scions correlates with enhanced plant vigour. Although we could not identify the direct effects of DNA methylation changes on gene expression (Fig. S8), two hetero-grafted scions displayed a similar expression profile, characterised by upregulation of genes involved in cell division and downregulation of genes involved in secondary metabolism and defence. Interestingly, a similar transcriptional change pattern is reported in hybrids for many heterotic plant species, and it is generally associated to an increase in plant vigour^27^. In the last decade, there has been a growing appreciation of the potential role of epigenetics in the molecular, cellular and developmental bases of heterotic vigour^28,29^. In previous studies performed in *Arabidopsis*, heterotic vigour was observed in the hybrid progeny obtained by crossing near-isogenic parents with variable epigenetic profiles^30,31^, indicating that the genetic difference in the parents is not the only factor triggering heterosis. In our study, we observed that a methylation decrease in the CHH context was associated to vigour in hetero-grafted eggplant scions, suggesting that changes in DNA methylation induced by the rootstocks in the scion can contribute to an increase in vigour. Importantly, a genome-wide decrease in CHH methylation was previously associated with hybrid vigour in *Arabidopsis*, and correlates with a general decrease of 24 nt siRNAs^29,32^. Nonetheless, plant DNA methylation in CHH context is considered to be highly stochastic^33^, and it is, therefore, remarkable to observe that DMRs have been found almost exclusively hypomethylated. However, previous studies performed on epigenetic hybrids showed that only a subset of DMRs can be directly associated with heterosis^31^, and we also found only little overlap among differentially express genes and DMRs in hetero-grafted vigorous scions. Therefore, it is possible that gene expression changes linked to enhanced vigour are not the direct consequence of most of the observed epigenetic alterations, and further studies performed at earlier developmental time points are necessary to identify epigenetic alterations potentially responsible to trigger vigour in grafted plants.

In hetero-grafted scions we observed upregulation of the methyltransferases *Smel*CMT3b and *Smel*MET (Fig. 3d). The corresponding Arabidopsis homologues genes *CMT3* and *MET1* encode for positive regulators of DNA methylation respectively at CHG and CG context^34,35^. By contrast, we did not observe significant changes in the expression of DNA methyltransferases associated to the CHH context (CMT2, DRM1 and DRM2 genes) (Table S8). Considering that in the same samples we also observed a genome-wide decrease in CHH methylation, while CHG and CG did not appear to be altered, it is possible that the overexpression of these factors can counterbalance a putative genome-wide demethylation tendency associated to the hetero-grafted scions. Nonetheless, *Smel*CMT3b gene is suppressed in eggplant during drought and salt stress, which are conditions associated with the inhibition of plant growth^21^, and the fact that this gene is upregulated in our hetero-grafted scions could suggest it is correlated with plant vigour.

Recent findings have shown that plant vigour induced by the disruption of the mitochondrial- and plastid-targeted protein (MSH1) function can be transmitted through a graft junction in Arabidopsis and tomato, and was also found to be involved in epigenetic regulation^36^. However, considering that the eggplant *MSH1* homologue (SMEL_009g333200.1) was not found differentially expressed in our analysis, it is very likely that the grafting-induced vigour we observed occurred independently of MSH1. Nonetheless, we also observed that none of the DNA demethylases annotated in eggplant appears to be differentially regulated in hetero-grafted scions at the time of analysis (Table S8). This could indicate that the lower CHH methylation observed is the result of passive demethylation processes, or alternatively, it is the consequence of active demethylation occurring at an earlier stage of development.

In plants, methylation in the CHH context is normally associated with suppression of TEs expression. Therefore, it is surprising that the observed genome-wide decrease of methylation in hetero-grafted scions does not correlate with a wide increase of TE expression (Fig. S10), but is rather associated with a more complex regulation resulting in many TEs being downregulated. One possible explanation is that hypomethylation might activate other silencing mechanisms to reduce RNA transcripts of potentially active TEs, for example, post-transcriptional gene silencing (PTGS) and this hypothesis is consistent with the preferential suppression of younger and potentially more active LTR-TEs. A similar age-dependent LTR-TEs regulation was also observed in a recent study performed on tomato epigenetic mutants, and was associated to the preferential methylation of young transposons by the DNA methyltransferase CMT3^37^.

Our work provides the first DNA methylome of eggplant and sheds new light on the molecular mechanisms underlying the effects of rootstock on the scion. Our data suggest the involvement of epigenetic regulation to control vigour of grafted Solanaceae species, showing that epigenetic changes (especially decrease in CHH methylation) correlate with vigour in two hetero-grafted eggplant combinations, mirroring the well-known effects reported for hybrids and epi-hybrids. In this context, we conclude that the use of grafting represents a promising alternative to traditional breeding to manipulate plant epigenomes and improve plant production.

## Materials and Methods

### Plant material and sampling

Plants selected for this work include an eggplant DH line derived from the commercial hybrid ‘Ecavi’ (Rijk Zwaan, Netherlands), wild *Solanum torvum* and tomato F_1_ *S. lycopersicum* x *S. habrochaites* hybrid ‘Emperador RZ’ (Rijk Zwaan, Netherlands)^15^. ‘Ecavi’ plants were used as self-grafted controls and as scions for the following rootstock/scion grafting combinations: ‘Ecavi’/’Ecavi’ (self-grafted control), ‘Emperador RZ’*/* ‘Ecavi’, *S. torvum/* ‘Ecavi’. Three biological replicates for each condition were considered in this experiment. Seeds were sterilised as described by Gisbert et al., 2006 and germinated in growth chambers under long-day conditions (26 °C, 16-h light, 8-h dark)^13^. Grafting was performed using the cleft methods described by Lee (1994)^13^ and moved in an experimental greenhouse of Carmagnola, Italy (44°53′N; 7°41′E) 3 months after grafting, during the 2017 growing season. Scion leaves of three biological replicates for each grafted and control plant type were sampled 5 months after grafting, flash-frozen in liquid nitrogen and stored at −80 °C. Those samples were used to perform all the genomic analyses (BS-seq, RNA-seq, qPCR) described below.

### Phenotypic evaluation

Three biological replicates of hetero-grafted (‘Emperador RZ’*/* ‘Ecavi’, *S. torvum/* ‘Ecavi’) and control plants (‘Ecavi’/’Ecavi’) have been phenotypically monitored every week in the experimental greenhouse and found consistent with previous phenotypic evaluation performed on the same grafting combinations^13,15^. Plant height and scion developmental architecture were annotated, and the sampling time was selected at the moment of the largest vigour difference between hetero-grafted and self-grafted combinations.

### Nucleic acid extraction

DNA and RNA were extracted from 100 mg of frozen leaves tissue collected from eggplant scions or ungrafted plants. Nucleic acids extraction was conducted using three biological replicates for each condition. For each sample, genomic DNA was extracted using the Qiagen Plant DNeasy kit (Qiagen, Hilden, Germany). Total RNA was extracted using the Spectrum Plant Total RNA Kit (Sigma, Saint Louis, USA) method according to the manufacturer’s instructions.

### Bisulfite conversion of genomic DNA

Genomic DNA (120 ng) were bisulfite-converted using the EZ DNA Methylation-Gold Kit (Zymo Research, Irvine, CA) following the manufacturer’s recommendation with minor modifications. In order to increase the chances to obtain a high conversion rate, the conversion step was repeated twice. Samples underwent the following reaction in a thermal cycler: 98 °C for 10 min, 64 °C for 2.5 h, 98 °C for 10 min, 53 °C for 30 min, then eight cycles at 53 °C for 6 min followed by 37 °C for 30 min and a final incubation at 4 °C overnight. Bisulfite conversion was performed on duplicates of each experimental condition.

### Library preparation and sequencing

Duplicates of converted samples were immediately used to prepare bisulfite libraries employing the TrueSeq DNA Methylation Kit (Illumina, San Diego, CA) accordingly to the protocol’s instruction. Libraries for RNA expression analysis were prepared in duplicates from 2 µg of total RNA using the TrueSeq Stranded mRNA Sample Prep Kit (Illumina, San Diego, CA) following the manufacturer’s instructions. Libraries quality and fragment sizes were checked with a TapeStation 2200 (Agilent Technologies, Santa Clara, CA) instrument and the DNA quantified by PCR on a LightCycler 480 II (Roche Molecular Systems, Pleasanton, CA) using the Library Quantification Kit (Roche Molecular Systems, Pleasanton, CA). Bisulfite and RNA-sequencing reactions were performed on a NextSeq500 using HighOutput chemistry, at the core facility of the Sainsbury Laboratory University of Cambridge (SLCU, Cambridge, UK).

### Sequencing processing

Whole genome bisulfite sequencing (WGBS) and RNA-seq raw reads were trimmed using Trimmomatic^38^ to remove adaptor sequences. For bisulfite libraries, high-quality trimmed sequences (on average 90,7% of raw reads) were aligned against the eggplant reference genome^25^ using Bismark^39^. Genome coverage was estimated taking into consideration the following parameters: length of reads, read numbers and eggplant genome size (~1.2 Gb). Not repeated DNA regions of the eggplant chloroplast sequence^40^ were used to estimate the bisulfite conversion. We computed chloroplast mappability on the eggplant genome using the gem‐mappability tool from the Gem library^41^, with a k-mer size of 75 bp and allowing a maximum of one mismatch, and only unique regions (mappability = 1) were used to estimate conversion. To account for non-converted DNA, we applied a correction according to a previously established pipeline^42^. Briefly, the number of methylated reads were decreased as m* = max(0, m – nc) (where m* is the corrected number of methylated reads, m is the raw number of methylated reads, *n* is the total number of reads and c is the conversion rate). The DNA methylation at different cytosine contexts were plotted on the chromosome using the R package *DMRcaller*^43^.

For transcript-level analysis, reads were mapped with TopHat^44^ on the eggplant reference genome^25^, using parameters–max-multihits 1–read-realign-edit-dist 0–no-mixed. Mapped reads were subsequently counted using htseq-count^45^ with parameters–order name–type = exon–stranded = reverse.

### Analysis of DNA methylation

DNA methylation analysis was performed accordingly to the previously established method^46^. Briefly, DMRs were defined using the R package *DMRcaller*^43^ between scions of control self-grafted replicates and each of the two hetero-grafted conditions (‘Emperador RZ’ or *S. torvum*). We compared cytosine methylation for the CHH contexts with the function computeDMRsReplicates, using the ‘bins’ method with a bin size of 300 nt, with 0.1 minimum methylation different, 2 minimum cytosine count, 4 minimum cytosine coverage and a minimum *p* value of 0.05. Annotated genes and repeats from the eggplant reference genome were used to extract genomic features. The Repeat Masker annotation^25^ was used to define the class and the family of each TEs. The overlap of DMRs in CHH context and genomics features were calculated in R with the *GenomicRanges* package^47^ and compared to the overlap found using the same amount of randomly generated regions (300 bp size) along the eggplant genome.

### De novo annotation of LTR-TEs

As most of the plant genomes are composed of TEs and LTRs are the most abundant, we developed the functional annotation pipeline *LTRpred*^26^ to de novo re-annotate retrotransposons within the eggplant genome assembly. The output of *LTRpred* was then used as input for htseq-count. We applied a stringent presence-call filter, restricting the analysis to those annotated genes or LTRs with more than five counts per million in the two biological replicates. Differential expression was assessed with DEseq^48^, using as thresholds log2 fold change >1 and a Benjamini–Hochberg’s FDR <0.05.

### GO enrichment analysis

We performed a GO enrichment analysis using ShinyGO online tool^22^ with Fisher’s exact test, false discovery rate (FDR) correction and selecting a 0.05 *p* value cut-off. This approach was employed for the analysis of statistically significant DE genes and LTR-TEs.

### Target expression analysis

RNA-seq data were validated using qPCRs for 11 genes up or downregulated according to FPKM values. For real‐time qRT‐PCR analysis, total RNA (2 μg) was treated with RQ1 DNase (Promega, Madison, Wisconsin) and reverse‐transcribed with the SuperScript VILO cDNA Synthesis Kit (Thermo Fisher, Waltham, Massachusetts), according to the manufacturer’s instructions. PCRs were carried out in biological duplicates and three technical replicates using 10 ng of template cDNA, 10 nM target‐specific primers (Table S5) and LightCycler 480 SYBR Green I Master (Roche Molecular Systems, Pleasanton, CA) in the LightCycler 480 II detection system (Roche Molecular Systems, Pleasanton, CA) in a volume of 10 μl. GADPH was used as a housekeeping gene.

## Supplementary information

Supplemental material

Supplemental Tables

## Data Availability

Sequencing data have been deposited in Gene Expression Omnibus under the accession number GSE136785.
