# Supplementary material for: Grafting vigour is associated with DNA de-methylation in eggplant
Source: Hortic Res. 2021 Nov 1;8:241. doi: 10.1038/s41438-021-00660-6 (PMC8558322; doi:10.1038/s41438-021-00660-6)
Supplement: Supplementary file 1 — Supplemental material [file 41438_2021_660_MOESM1_ESM.pdf]

Supplementary Material

Supplementary tables legends

Supplementary Table S1. Metrics of WGBS analysis

Supplementary Table S2. Differentially methylated regions found in TOR vs Self conditions

Supplementary Table S3. Differentially methylated regions found in EMP vs Self conditions

Supplementary Table S4. Metrics of RNA-seq analysis

Supplementary Table S5. List of up-regulated genes in the two heterografted conditions (i.e. EMP and TOR; *P*<0.05).

Supplementary Table S6. List of down-regulated genes in the two heterografted conditions (i.e. EMP and TOR; *P*<0.05).

Supplementary Table S7. List of primers used for RNA-seq validations

Supplementary Table S8. Eggplant DNA methyltransferases and demethylases gene IDs and their level of transcription in EMP and TOR plants. Statistical significant p values (<0.05) are highlighted. NA = not analysed/not significant/not expressed.

Supplementary Table S9. Output of enrichment analysis on up-regulated genes in *S. torvum* and 'Emperador RZ'. The analysis was conducted using a p-value cut-off of 0.05.

Supplementary Table S10. Output of enrichment analysis on down-regulated genes in *S.torvum and* 'Emperador RZ'. The analysis was conducted using a p-value cut-off of 0.05.

Supplementary Table S11. List of transposable elements differentially expressed in at least one hetero-grafted condition (pvalue < 0.05)

Supplementary Table S12. Number of elements belonging to different transposable elements classes in *S.torvum* and '*Emperador R*Z'.

Supplementary Table S13. List of up-regulated LTRs in the two heterografted conditions (i.e. EMP and TOR; *P*<0.05).

Supplementary Table S14. List of down-regulated LTRs in the two heterografted conditions (i.e. EMP and TOR; *P*<0.05).

Supplementary Figure Legends

Figure S1. DNA methylation profile in eggplant leaves of not grafted plants. DNA methylation content in CG (red), CHG (blue) and CHH (yellow) contexts, represented in the 12 eggplant chromosomes. In dashed black is reported the gene density.

Figure S2. DNA methylation profiles in self-grafted and hetero-grafted eggplant. Distribution of DNA methylation at each cytosine context (mCG, mCHG and mCHH) along chromosomes 2 to 12, in eggplant scions self-grafted (Self), or grafted onto *S. torvum* (TOR) or tomato 'Emperador RZ' (EMP) rootstocks. The profiles in chromosome 1 are displayed in **Figure 2**.

Figure S3. **DNA methylation distribution at the main TEs classes.** Distribution of averaged DNA methylation (at CG, CHG and CHH contexts) at annotated TEs of main three groups in eggplant genome, in eggplant scions self-grafted (Self), grafted on *S. torvum* (TOR) or tomato 'Emperador RZ' (EMP).

Figure S4. DMR analysis. a Identified CHH DMRs in TOR and in EMP, using self-grafted scions replicates as common denominator. **b** Distribution of CHH DMRs in scions grafted on EMP rootstock compared to self-grafted scions. DMR density (axes on the left) in 5 million bp windows is plotted for each chromosome. For reference, the density of repeats and coding genes are also plotted (axes on the right). **c** Distribution of CHH DMRs in scions grafted on TOR rootstock compared to self-grafted scions. Description is as in b. **d** Proportion of CHH DMRs in TOR (N = 9,745) and in EMP (N = 16,304) that overlap with genomics features in comparison to randomly selected 300bp regions (Control). Promoters are defined as the DNA sequence located 1Kb upstream of each annotated gene. Significant enrichment calculated with one-tailed Fisher's exact test is marked with *** (p-value < 10^{-100}) and ** (p-value < 10^{-18}).

Figure S5. Overlap among differentially expressed annotated genes and LTR-TEs. Venn-diagrams displaying up-regulated (**a**) and down-regulated (**b**) genes, or up-regulated (**c**) and down-regulated (**d**) annotated LTR-TEs, in eggplant scions grafted onto *S. torvum* (TOR) and tomato 'Emperador RZ' (EMP) compared to self-grafted control.

Figure S6. Validation of RNA-seq expression data. RNA accumulation was measured with qPCR for a subset of 11 genes randomly selected with |log2FC| > 3. Bars represent up/down- regulated genes in eggplant

scions grafted onto *S. torvum* (**a**) or tomato ‘Emperador RZ’ (**b**) rootstocks compared to self-grafted plants (Self). Both up-regulated (top line) and down-regulated (bottom line) genes were tested. Error bars represent SD of three replicates.

Figure S7. Gene enrichment analysis in self-grafted and hetero-grafted eggplants. Lists of enriched Gene ontology categories (with a p-value < 0.05) of differentially expressed genes shared in hetero-grafted compared to self-grafted scions. Different graphs display information for down-regulated (**a**) and up-regulated (**b**) genes. The number of genes belonging to each GO category is reported at the top of each bar. The specific p-values found for the enrichment of each GO category is reported in Tables S9 and S10.

Figure S8. Overlap among DMRs and differentially expressed genes. Venn-diagrams displaying promoters (1Kb DNA sequence located upstream of each gene) and coding sequences (CDS) from differentially expressed genes (DEGs) or overlapping DMRs in EMP (**a**,**b**) or in TOR conditions (**c**,**d**).

Figure S9. Hetero-grafted scions display similar LTR-TEs expression profiles. a Heatmap illustrating the expression (log2 FPKM) of differentially expressed *de novo*-annotated LTR TEs (|log2| > 2 and FDR <0.05) in hetero-grafted plants and self-grafted controls. Each row represents one LTR TE (*n* = 63). Coloured bars on the top-left indicate the expression level. Each column represents an eggplant scion grafted on different rootstock (TOR = *S. torvum*, EMP = tomato ‘Emperador RZ’ and Self = self-grafted). Expression values were ordered according to hierarchical clustering (hclust and heatmap3 R software environment). The Euclidean distance dendrogram is presented on the left. **b** Scatter-plots of annotated LTR-TEs (*n* =6,583) in Self and EMP conditions, each dot represents a gene and in red are displayed differentially expressed LTR-TEs between the two conditions. **c** Scatter-plots of annotated LTR-TEs (*n* =6,583) in Self and TOR conditions. In purple are displayed differentially expressed LTR-TEs between the two conditions.

Figure S10. CHH hypomethylation in differentially expressed LTR-TEs. Box plots displaying the averaged mCHH methylation on downregulated (DOWN) and upregulated (UP) LTR-TEs in EMP (**a**) and in TOR (**b**) conditions, compared to self-grafted scions (self). Data are from both replicates.

Figure S1

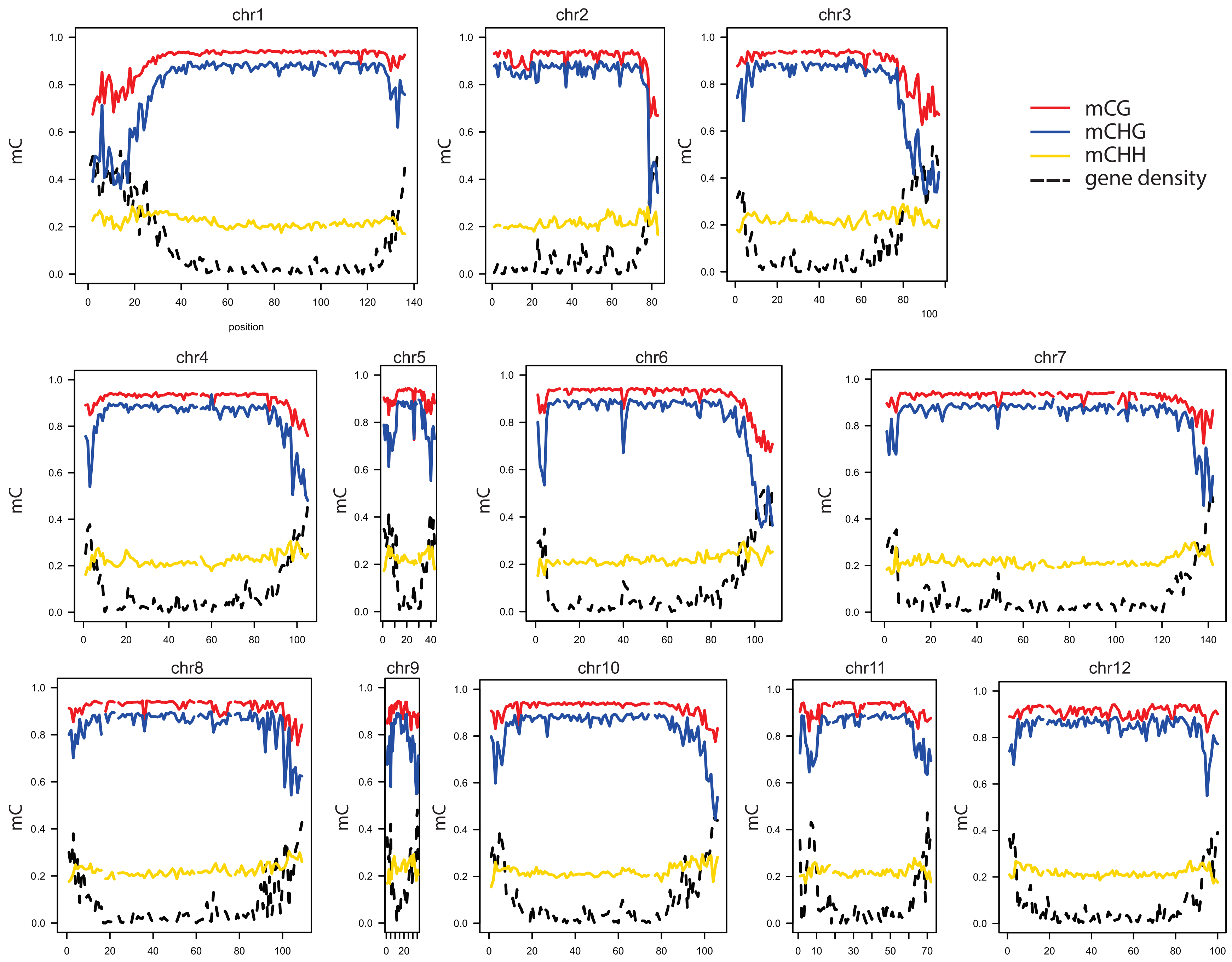
chr1
chr2
chr3
chr4
chr5
chr6
chr7
chr8
chr9
chr10
chr11
chr12
mC
position
mCG
mCHG
mCHH
gene density

Figure S2

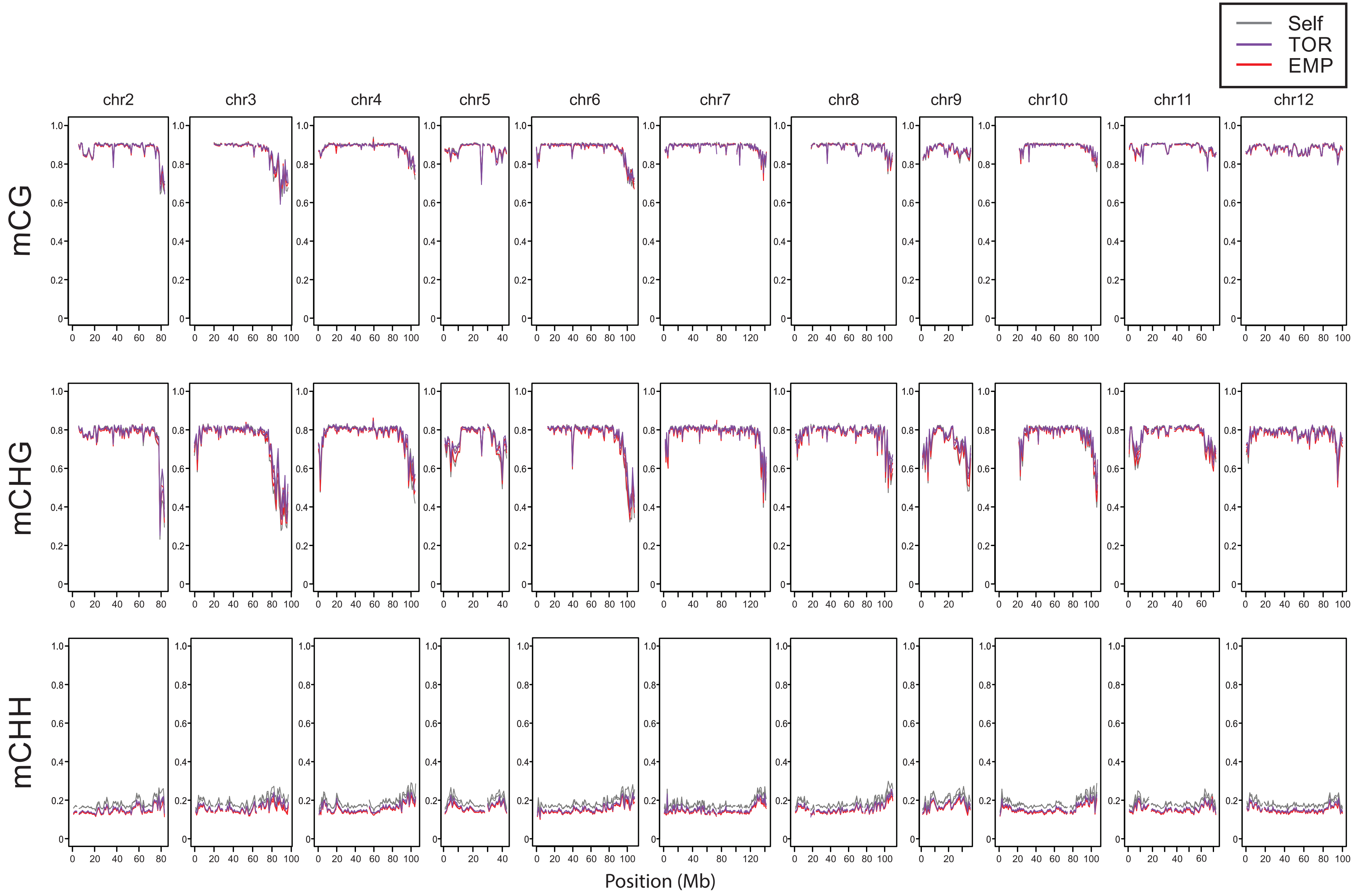
Self
TOR
EMP
chr2
chr3
chr4
chr5
chr6
chr7
chr8
chr9
chr10
chr11
chr12
mCG
mCHG
mCHH
Position (Mb)

Figure S3

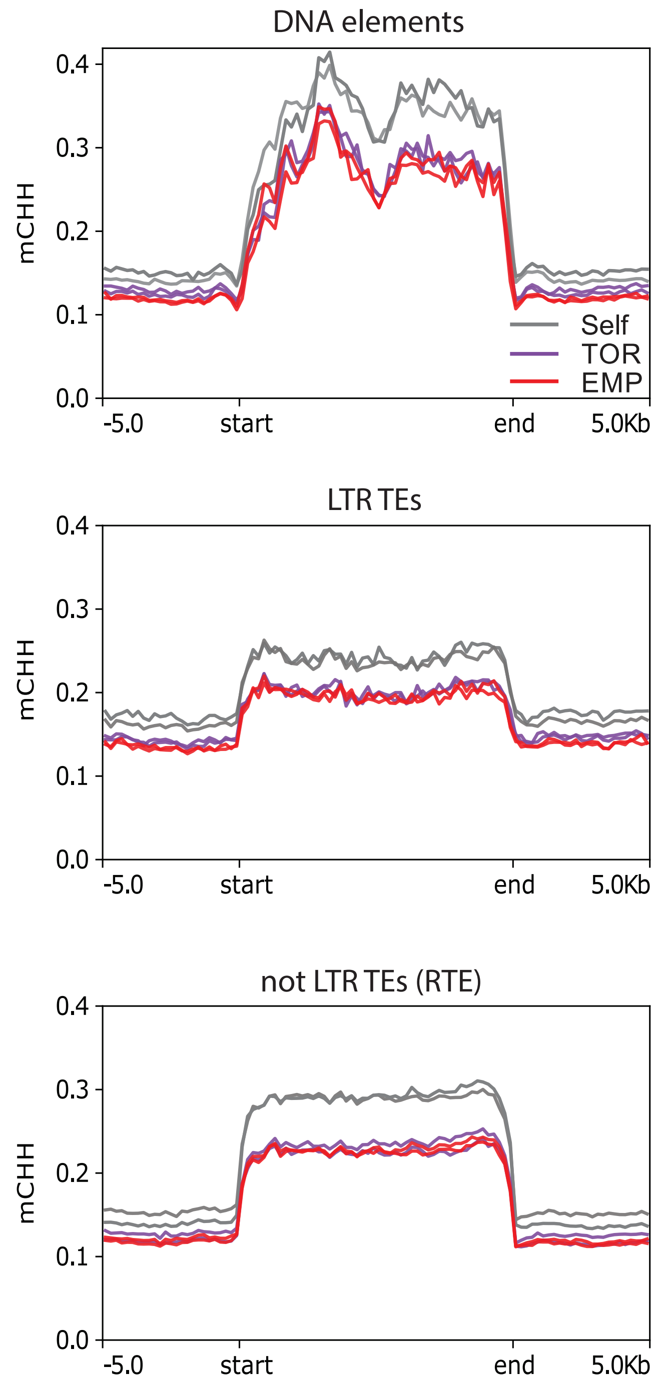
DNA elements
mCHH
0.4
0.3
0.2
0.1
0.0
Self
TOR
EMP
-5.0
start
end
5.0Kb
LTR TEs
mCHH
0.4
0.3
0.2
0.1
0.0
-5.0
start
end
5.0Kb
not LTR TEs (RTE)
mCHH
0.4
0.3
0.2
0.1
0.0
-5.0
start
end
5.0Kb

Figure S4

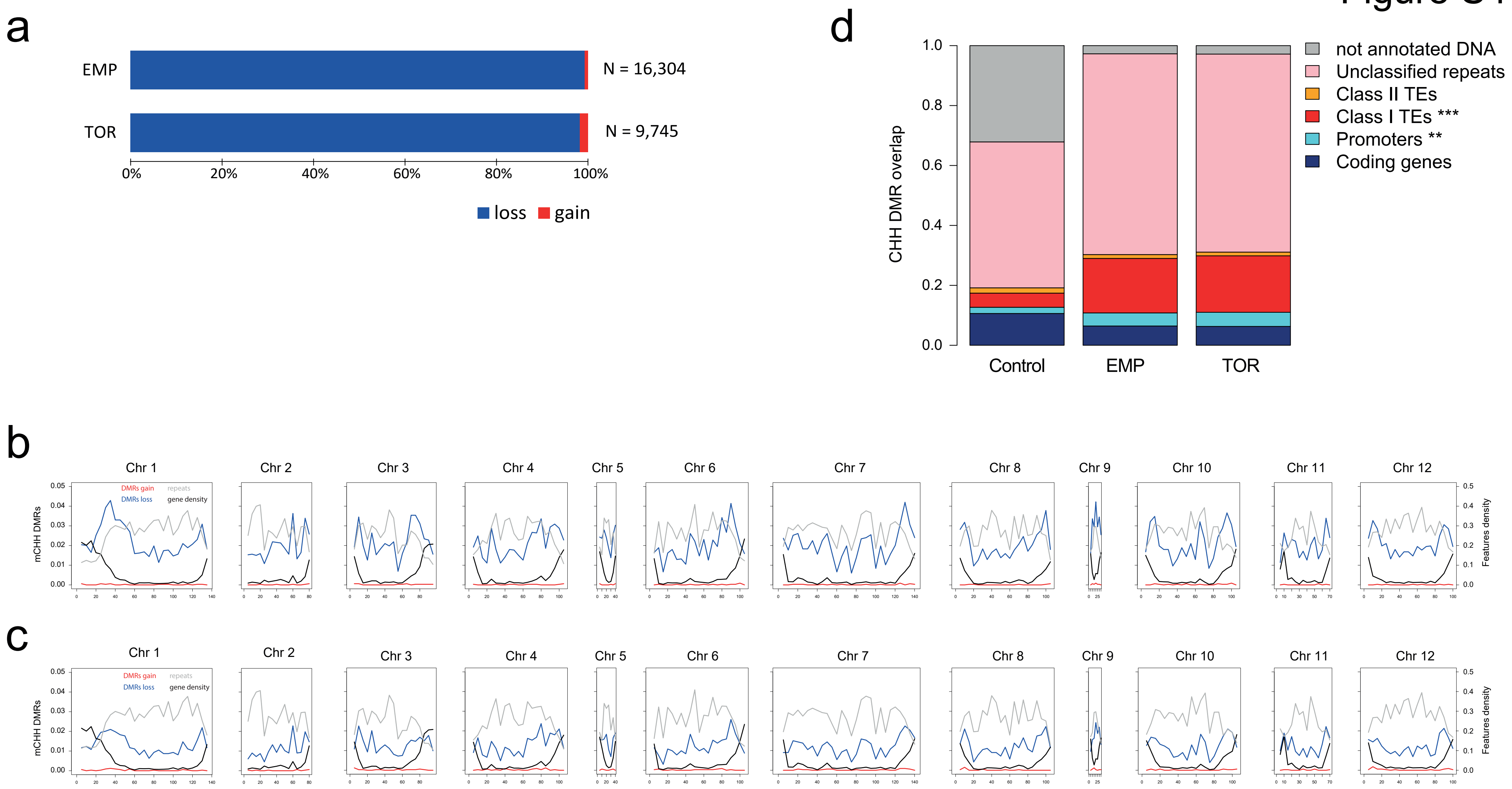

a
EMP
N = 16,304
TOR
N = 9,745
0%
20%
40%
60%
80%
100%
loss
gain
d
CHH DMR overlap
1.0
0.8
0.6
0.4
0.2
0.0
Control
EMP
TOR
not annotated DNA
Unclassified repeats
Class II TEs
Class I TEs ***
Promoters **
Coding genes
b
Chr 1
Chr 2
Chr 3
Chr 4
Chr 5
Chr 6
Chr 7
Chr 8
Chr 9
Chr 10
Chr 11
Chr 12
mCHH DMRs
DMRs gain
DMRs loss
repeats
gene density
Features density
c
Chr 1
Chr 2
Chr 3
Chr 4
Chr 5
Chr 6
Chr 7
Chr 8
Chr 9
Chr 10
Chr 11
Chr 12
mCHH DMRs
DMRs gain
DMRs loss
repeats
gene density
Features density

Figure S5

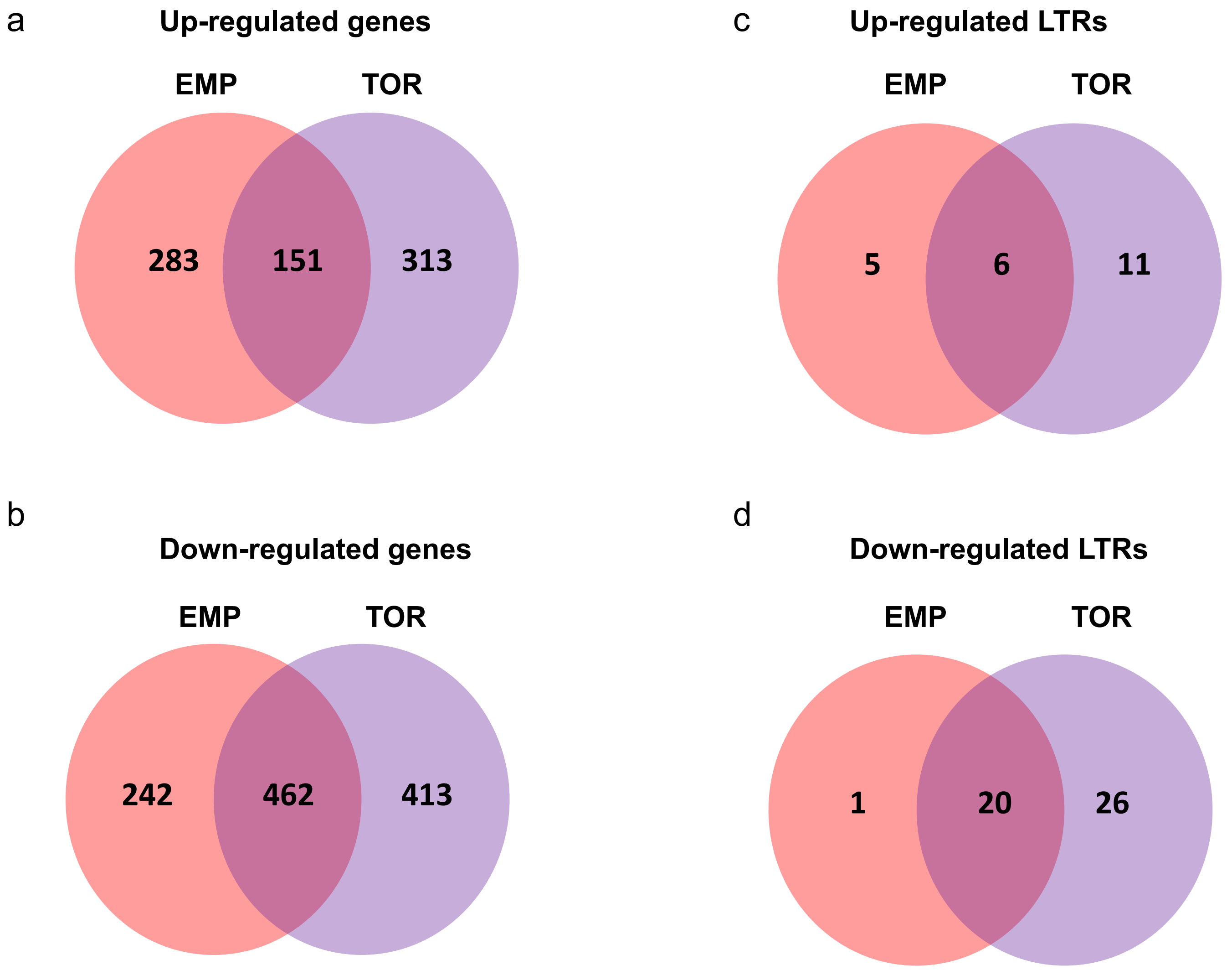
a
Up-regulated genes
EMP
TOR
283
151
313
c
Up-regulated LTRs
EMP
TOR
5
6
11
b
Down-regulated genes
EMP
TOR
242
462
413
d
Down-regulated LTRs
EMP
TOR
1
20
26

Figure S6

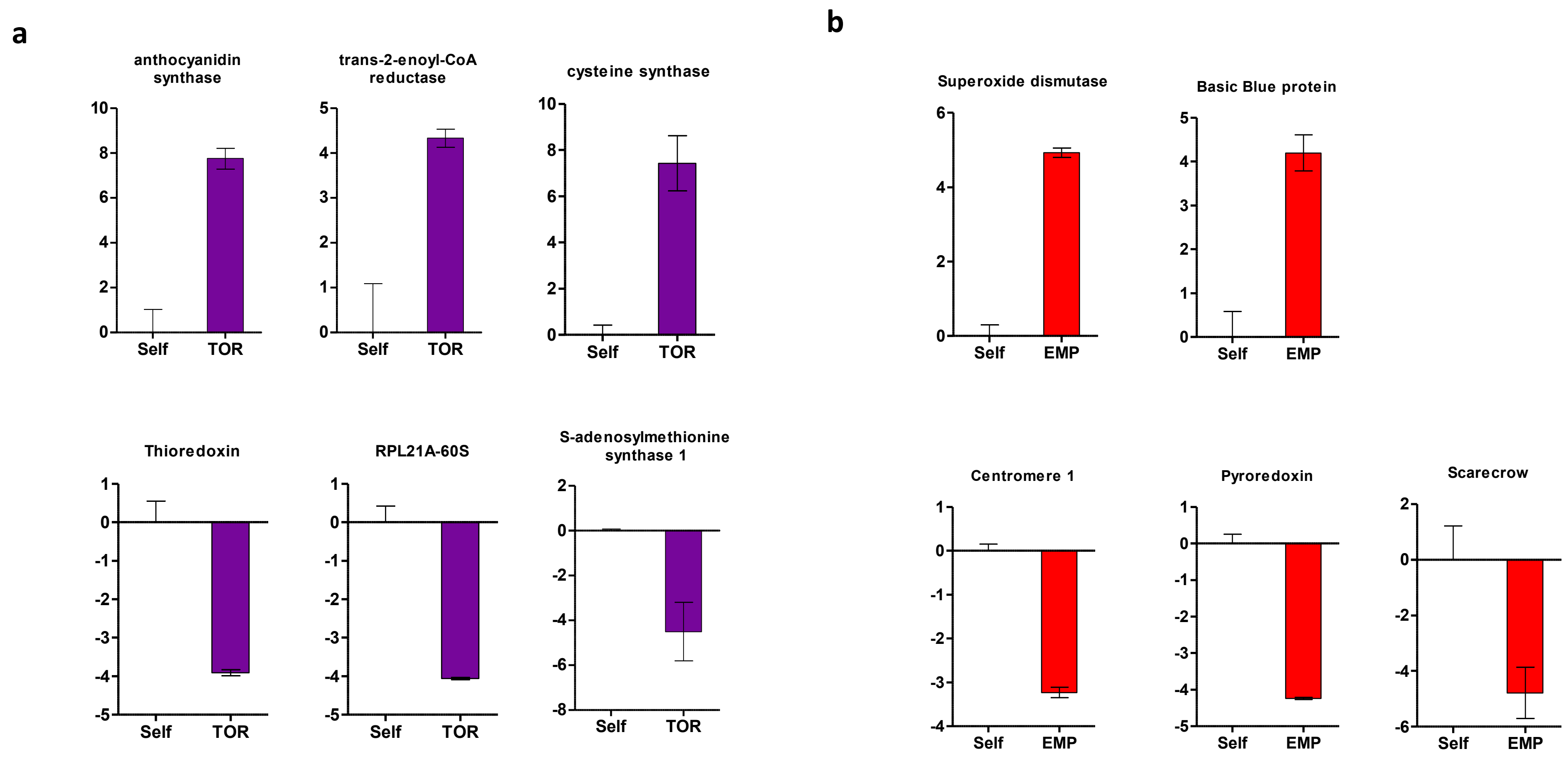
a
anthocyanidin synthase
10
8
6
4
2
0
Self
TOR
trans-2-enoyl-CoA reductase
5
4
3
2
1
0
Self
TOR
cysteine synthase
10
8
6
4
2
0
Self
TOR
Thioredoxin
1
0
-1
-2
-3
-4
-5
Self
TOR
RPL21A-60S
1
0
-1
-2
-3
-4
-5
Self
TOR
S-adenosylmethionine synthase 1
2
0
-2
-4
-6
-8
Self
TOR
b
Superoxide dismutase
6
4
2
0
Self
EMP
Basic Blue protein
5
4
3
2
1
0
Self
EMP
Centromere 1
1
0
-1
-2
-3
-4
Self
EMP
Pyroredoxin
1
0
-1
-2
-3
-4
-5
Self
EMP
Scarecrow
2
0
-2
-4
-6
Self
EMP

Figure S7

a

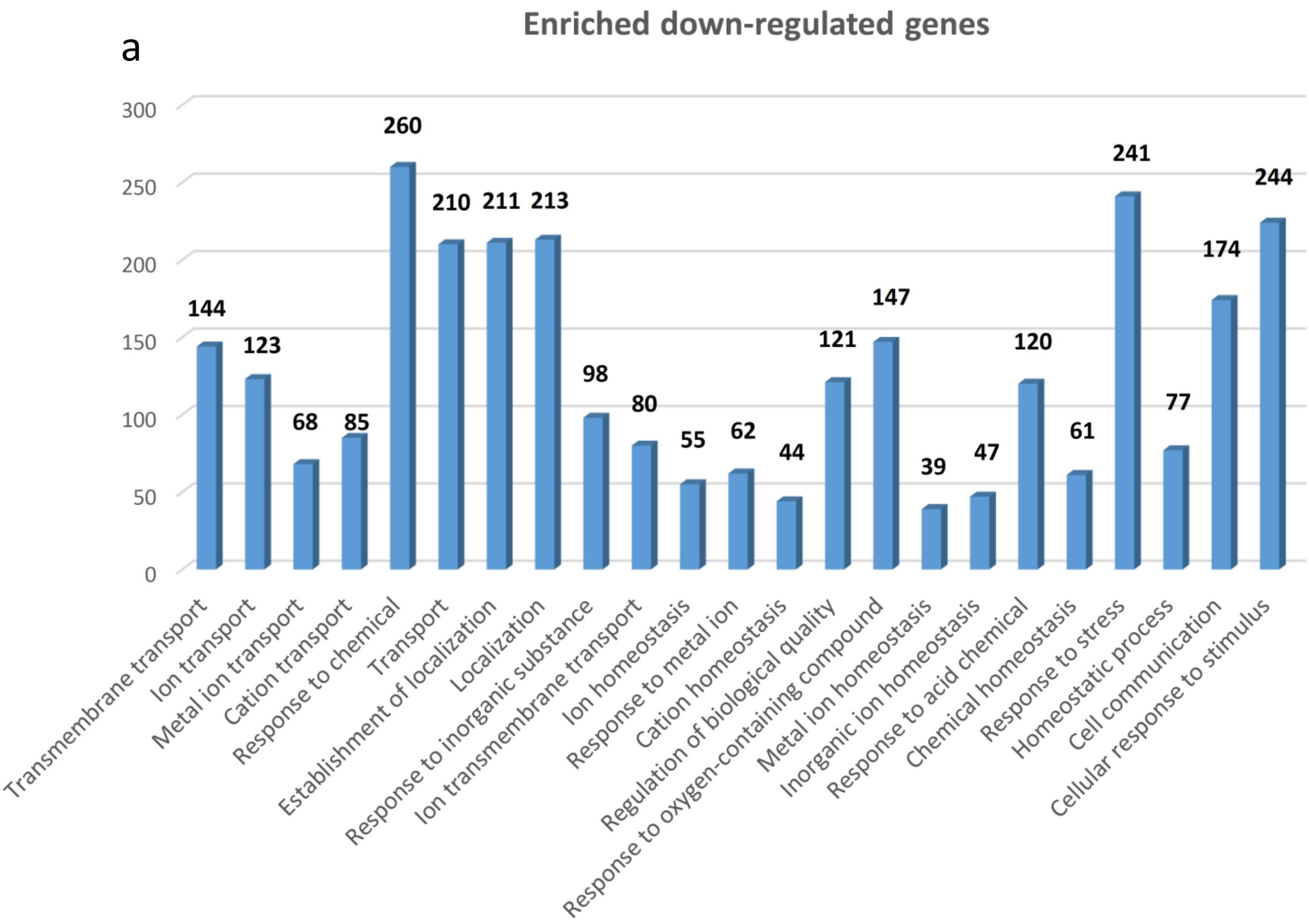

Enriched down-regulated genes
300
250
200
150
100
50
0
144
123
68
85
260
210
211
213
98
80
55
62
44
121
147
39
47
120
61
241
77
174
244
Transmembrane transport
Ion transport
Metal ion transport
Cation transport
Response to chemical
Transport
Establishment of localization
Localization
Response to inorganic substance
Ion transmembrane transport
Ion homeostasis
Response to metal ion
Cation homeostasis
Regulation of biological quality
Response to oxygen-containing compound
Metal ion homeostasis
Inorganic ion homeostasis
Response to acid chemical
Chemical homeostasis
Response to stress
Homeostatic process
Cell communication
Cellular response to stimulus

b

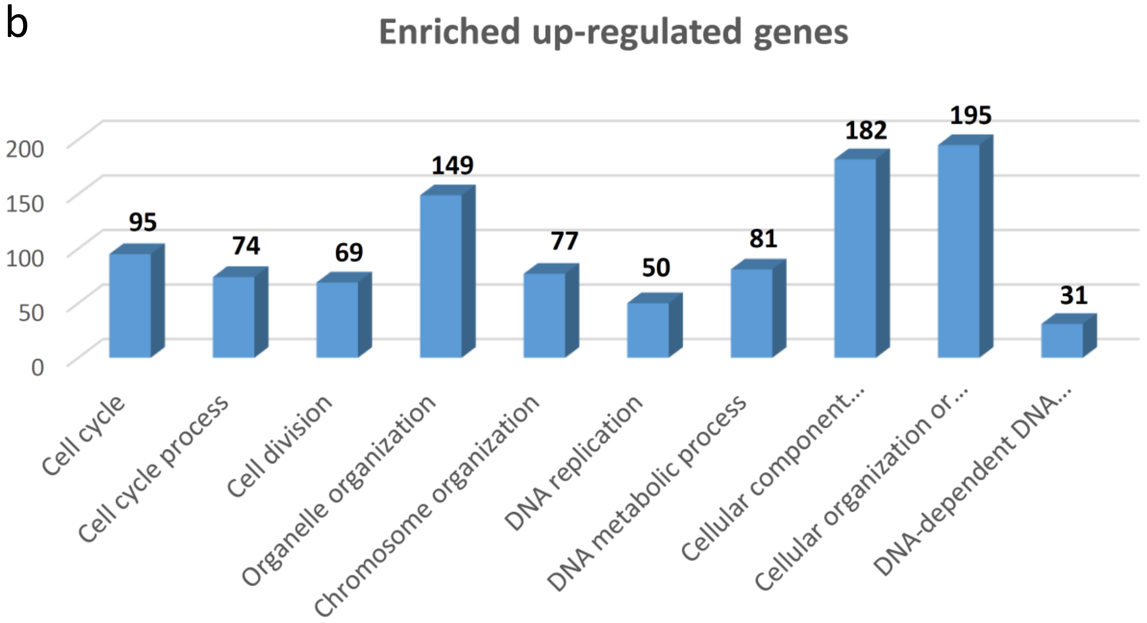

Enriched up-regulated genes
200
150
100
50
0
95
74
69
149
77
50
81
182
195
31
Cell cycle
Cell cycle process
Cell division
Organelle organization
Chromosome organization
DNA replication
DNA metabolic process
Cellular component...
Cellular organization or...
DNA-dependent DNA...

Figure S8

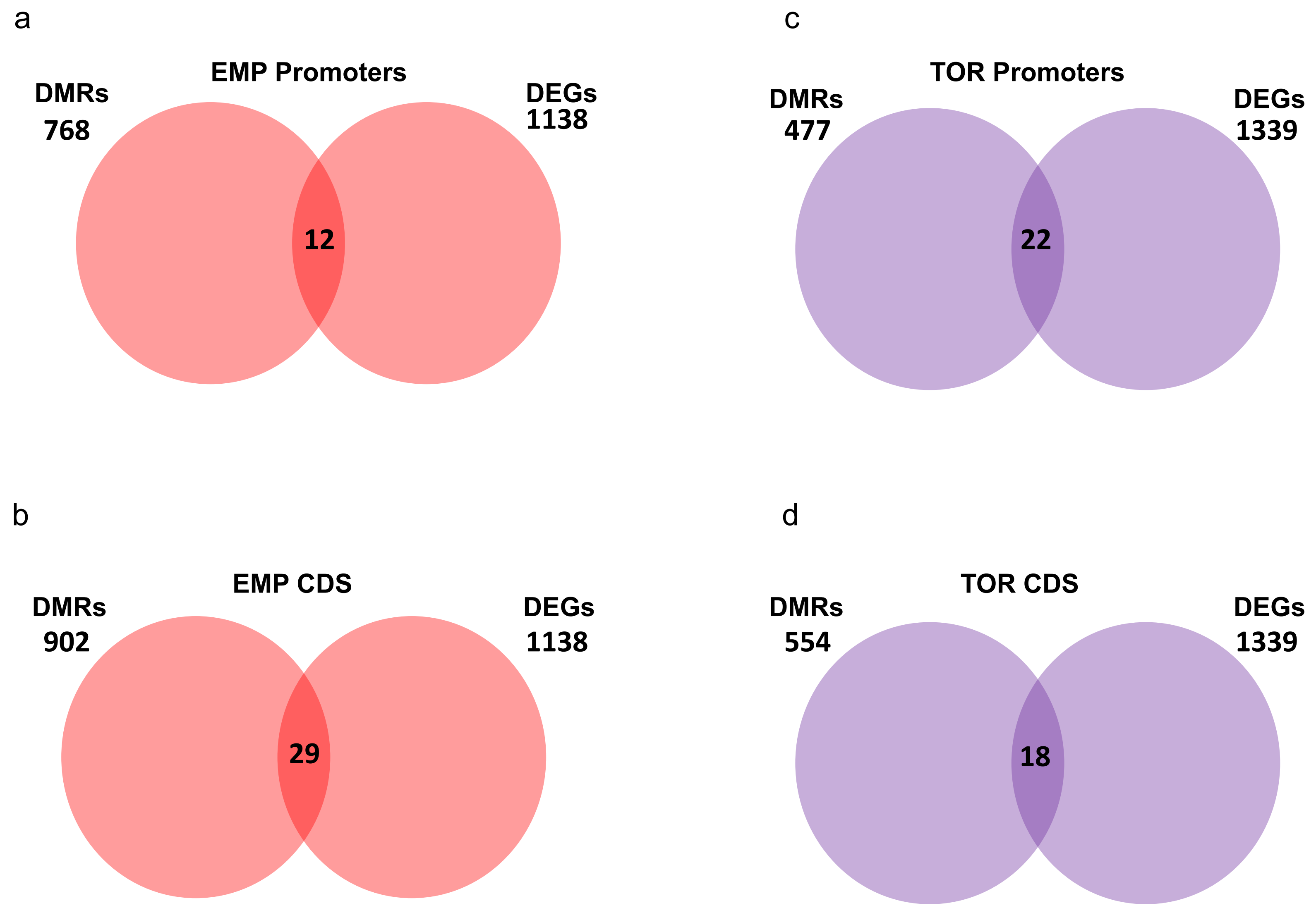

a
EMP Promoters
DMRs
768
DEGs
1138
12
b
EMP CDS
DMRs
902
DEGs
1138
29
c
TOR Promoters
DMRs
477
DEGs
1339
22
d
TOR CDS
DMRs
554
DEGs
1339
18

Figure S9

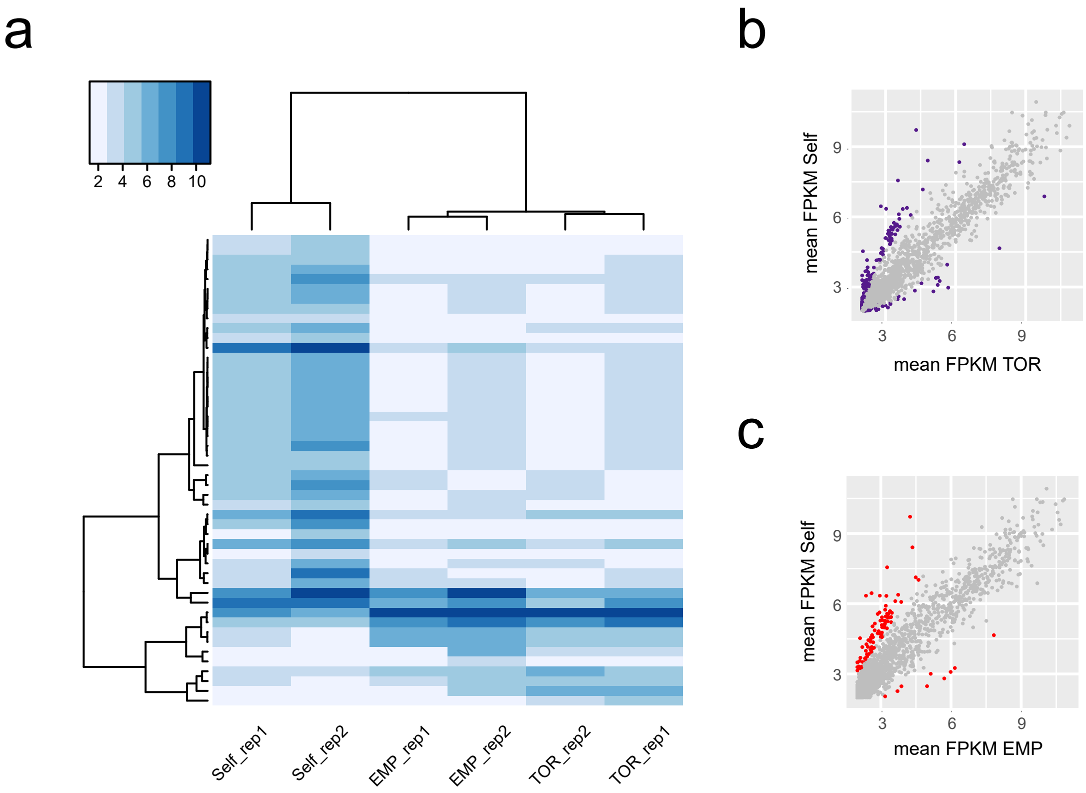
a
2
4
6
8
10
Self_rep1
Self_rep2
EMP_rep1
EMP_rep2
TOR_rep2
TOR_rep1
b
mean FPKM Self
mean FPKM TOR
3
6
9
c
mean FPKM Self
mean FPKM EMP
3
6
9

Figure S10

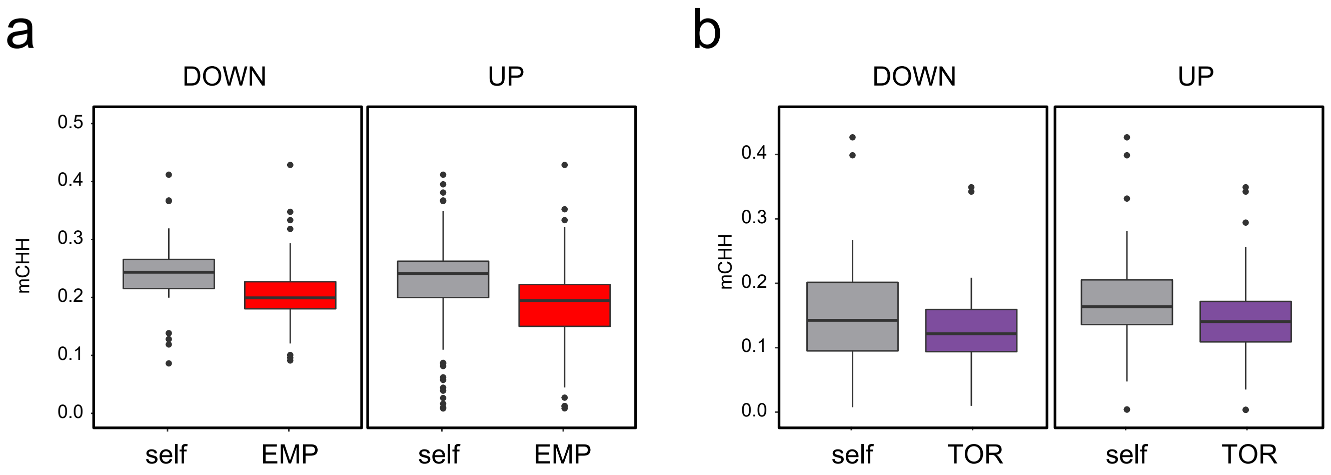
a
DOWN
UP
mCHH
0.5
0.4
0.3
0.2
0.1
0.0
self
EMP
self
EMP
b
DOWN
UP
mCHH
0.4
0.3
0.2
0.1
0.0
self
TOR
self
TOR
